# METABOLOMIC PROFILES OF DEMENTIA RISK AND MOBILITY DECLINE IN A BIRACIAL COHORT OF OLDER ADULTS

**DOI:** 10.1093/geroni/igad104.3723

**Published:** 2023-12-21

**Authors:** Qu Tian, Shanshan Yao, Luigi Ferrucci, Venkatesh Murthy, Ravi Shah, Anne Newman

**Affiliations:** National Institute on Aging, Baltimore, Maryland, United States; University of Pittsburgh School of Public Health, Pittsburgh, Pennsylvania, United States; National Institute on Aging, Baltimore, Maryland, United States; University of Michigan, Ann Arbor, Michigan, United States; Vanderbilt University Medical Center, Nashville, Tennessee, United States; University of Pittsburgh, Pittsburgh, Pennsylvania, United States

## Abstract

Age-related mobility decline precedes future cognitive impairment and dementia. Blood biomarkers underlying this connection are less clear. This study aims to examine metabolomic profiles of dementia risk and mobility decline in the Health, Aging and Body Composition Study. Dementia diagnosis was determined by race-specific decline in Modified Mini-Mental State Examination scores, medication use, and hospital records. 2450 participants were free of dementia at year 2 and had 613 targeted plasma metabolites. 534(22%) developed dementia during a mean follow-up of 9.3(SD=4.1) years. 2028 participants had repeated 20-meter usual gait speed up to 10 years follow-up. We performed Cox regression and linear regression with LASSO models to determine which metabolites would predict dementia risk and gait speed decline, respectively. After adjustment for age, sex, race, and education, 30 metabolites were significant predictors of incident dementia. Top-ranked metabolites included glyceric acid, niacinamide, androsterone 3- glucuronide, phosphatidylcholine (P-34:4)/PC(O-34:5), aminobutyric acid, xanthine, sphingomyelin (d18:1/14:0) (hazard ratios< 1), suberic acid, lysophosphatidylethanolamine (18:1), threitol, and andrenic acid (hazard ratios>1). These metabolites were enriched in arginine biosynthesis, alanine, aspartate and glutamate metabolism, glyoxylate and dicarboxylate metabolism and arginine and proline metabolism(p< 0.05). Of these metabolites, threitol, sphingomyelin (d18:1/14:0), adrenic acid, and phosphatidylethanolamine (P-36:0)/PE(O-36:1) were also significant predictors of mobility decline in a consistent direction. This longitudinal study reveals unique and shared metabolomic risk profiles for dementia and mobility decline. Several metabolites in amino-acid pathways and urea may be important in the development of dementia and certain sugar and lipids with long-chain and very long-chain fatty acids also predict mobility decline.

